# Development of a Smart Clinical Bluetooth Thermometer Based on an Improved Low-Power Resistive Transducer Circuit

**DOI:** 10.3390/s22030874

**Published:** 2022-01-24

**Authors:** Sitong Sun, Jinglun Xv, Wilson Wang, Chengyuan Wang

**Affiliations:** 1 School of Automation and Electronic Engineering, Qingdao University of Science and Technology, Qingdao 266000, China; 2019040020@mails.qust.edu.cn (J.X.); 2018040016@mails.qust.edu.cn (C.W.); 2 Department of Mechanical Engineering, Lakehead University, Thunder Bay, ON P7B 5E1, Canada; wilson.wang@Lakeheadu.ca

**Keywords:** smart sensors, clinical Bluetooth thermometers, resistive transducer, low-power circuit

## Abstract

Smart sensors have been used in many engineering monitoring and control applications. This work focuses on the development of a new type of clinical Bluetooth thermometer, based on an improved low-power resistive transducer circuit. Most existing resistive transducers use relatively complicated circuits with higher cost and power consumption. To tackle these problems, especially in real applications, an improved low-power resistive transducer circuit is proposed in this work and is used to develop smart Bluetooth thermometers. The parameters of the resistive transducer circuit are selected by quantitative analysis and optimization to improve the performance of the low-power resistive transducer circuit. The effectiveness of the proposed design technology was verified by tests. The temperature measurement error of the new smart Bluetooth thermometer is less than 0.1 °C, which can not only meet the clinical use requirements but also has lower cost and power consumption.

## 1. Introduction

**Figure 1:**
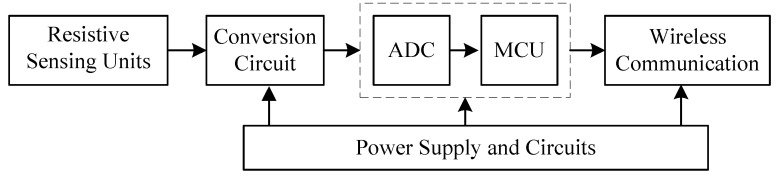
The composition of a typical smart sensor.

Smart transducers/sensors have been used in different engineering applications in manufacturing, system condition monitoring, automatic control, etc. [1,2]. Different from traditional transducers, a smart sensor has built-in functions, such as sensing, preconditioning, detection, monitoring, and information processing, in which a microcontroller unit (MCU) is used as the core processing unit. As illustrated in Figure 1, the outputs from the sensing units are amplified, adjusted, and then converted to digital signals, which are then fed to the MCU for processing. The digital signals can be sent to the receiver by proper wireless communication.

According to the measurement principle, the transducers or sensing units could be divided into an inductive mode, capacitive mode, and resistive modes [3]. The inductive transducer uses electromagnetic induction to convert the measured physical quantity into an inductance signal. A capacitive transducer uses a capacitor as a sensing element to convert the measured physical quantity into the change in capacitance. A resistive transducer is a sensing unit to convert the measured physical quantity into the change of resistance values [4]. This work will focus on resistive transducers because of their relatively small size, light weight, simple structure, and fast response speed [5,6].

Currently, the bridge circuit is commonly used in the conversion circuit in the preprocessing unit of smart transducers [7,8,9]. For example, a bridge circuit was used in the development of a textile pressure sensor in a classroom environment [10], which was installed in the fingertips of a glove to measure the pressure variation of each finger when holding an object; the measurement results showed that such a sensor could differentiate the steady-state and the impulse response of a system. In [11], a wearable plantar pressure monitoring system was developed for smart socks, in which a matrix bridge circuit was applied in sock pressure measurement. The pressure sensing matrix was made of conductive fabric and flexible piezo-resistive material [12]. The sensed foot pressure values were digitized and stored in the memory of the sensor-tag, which were then used to monitor the relative pressure of the foot. A non-contact monitoring/inspection smart phone was proposed in [13], based on the vibration characteristics of a beam subjected to thermal stress; a bridge circuit was used for temperature measurement. A simplified PTC circuit was proposed in [14]. It used a dividing voltage with a PTC resistor and fixed resistor to construct a conversion circuit, but it also required an operational amplifier (Op-amp) and many other components in the circuit. A fixed resistor grounding bleeder circuit was proposed in [15]; because it used a fixed resistor on the ground, its power consumption was relatively high (as discussed in Section 2.2.1). Although the aforementioned research results could realize the operation functions, their conversion circuits are either relatively complicated with higher costs or high power consumption, which may not be suitable for battery-powered products [16,17].

The objective of this work was to develop a new type of clinical Bluetooth thermometer, based on an improved low-power resistive transducer circuit. This product will be used for real-time human body temperature monitoring. The following novel aspects are provided in this study: (1) an improved low-power resistive transducer circuit is proposed to reduce cost and power consumption in smart sensor product development; (2) a quantitative method is suggested to optimize the system parameters based on the resolution of the minimal convention circuit and current loss analysis; and (3) a new type of Bluetooth thermometer product is developed for clinical applications. The effectiveness of the proposed circuit and the Bluetooth clinical thermometer was verified by systematic simulation tests.

The remainder of this paper is organized as follows: Section 2 explains the circuit principle of the resistive transducer circuit, and discusses the quantitative optimization of the circuit. Section 3 describes the development of the smart clinical thermometer based on the improved transducer conversion circuit. The effectiveness of the developed smart thermometer is examined using experimental and simulation tests in Section 4.

## 2. The Improved Low-Power Resistive Transducer Circuit

### 2.1. Bridge Conversion Circuitry

**Figure 2:**
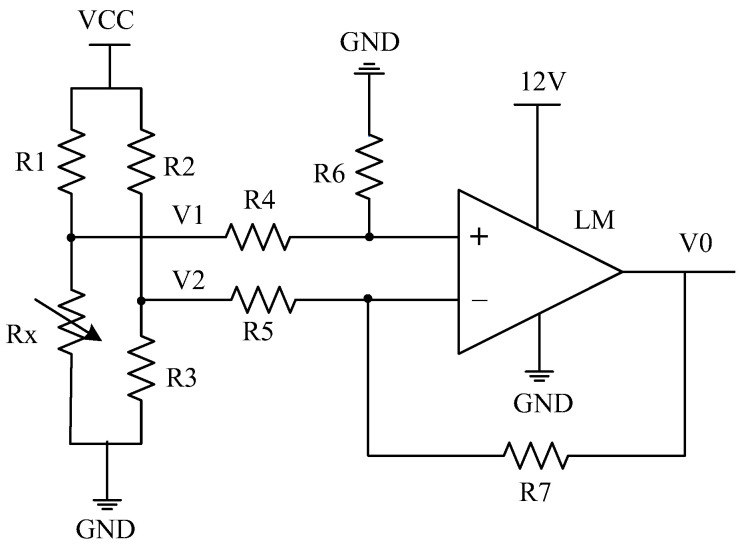
A classical bridge conversion circuit. *LM* is an Op-amp, where “+” and “−” represent the corresponding noninverting and inverting inputs, respectively.

The traditional resistive transducer circuit is mainly based on the use of a bridge conversion circuit [7,8,9], which consists of four resistors: *R*_1_, *R*_2_, *R*_3_, and *R*_X_, as illustrated in Figure 2. The resistance values of *R*_1_, *R*_2_, and *R*_3_ are fixed, where *R*_X_ is the resistive transducer (sensing unit) and *LM* is an Op-amp.

As illustrated in Figure 2, if the power supply (VCC) is $V_{CC}$, the voltage divider values *V*_1_ and *V*_2_ will be:

(1) $$V_{1}=\frac{R_{X}}{R_{1}+R_{X}}\times V_{CC}$$

(2) $$V_{2}=\frac{R_{3}}{R_{2}+R_{3}}\times V_{CC}$$

If *V*_1_ varies with the change of the variable resistor *R_X_* and *V*_2_ is fixed, the dropout voltage Δ*V* becomes:

(3) $$\Delta V=V_{1}-V_{2}$$

With the inputs *V*_1_ and *V*_2_, the Op-amp output *V*_0_ should cover the reference voltage range of the ADC to improve the processing accuracy. For the circuit in Figure 2, if $R_{4}=R_{5}$*, R*_6_ *= R*_7_*,* the amplification gain of the Op-amp will be *A =* R7R5, then:

(4) $$V_{0}=\Delta V\times A$$

The amplified voltage $V_{0}$ is inputted to the ADC. If the ADC has *N* bits, with the reference voltage of *V_ADC_* and output *D_o_*, from Equations (1)–(4), the resistance value of *R_X_* can be determined by:

(5) $$\left(\frac{R_{X}}{R_{1}+R_{X}}-\frac{R_{3}}{R_{2}+R_{3}}\right)\times V_{CC}\times A=\left(\frac{D_{o}}{2^{N}-1}\right)\times V_{ADC}$$

When the bridge is balanced or *V*_0_ = 0, the measured value of the ADC will be 0. Therefore, in theory, the measurement range of the bridge conversion circuit can cover the whole conversion range of the ADC [18,19]. For example, if the resistance range is 18~51 kΩ, when the resistance is 18 kΩ, the bridge is balanced. When the resistance is 51 kΩ, the measured value of the ADC is close to its full range, or:

(6) $$\frac{R_{1}}{1.8\times 10^{4}}=\frac{R_{2}}{R_{3}}$$

(7) $$\left(\frac{5.1\times 10^{4}}{R_{1}+5.1\times 10^{4}}-\frac{R_{3}}{R_{2}+R_{3}}\right)\times V_{CC}\times A=V_{ADC}$$

Given one resistance of *R*_1_, *R*_2_, and *R*_3_, the other two can be obtained from Equations (6) and (7). It can be seen that the bridge conversion circuit is complex with a higher cost.

### 2.2. The Minimal Convention Circuit

The minimal convention circuit consists of components, such as a DC power supply, grounding, a fixed resistor, and a resistive transducer. Its output is directly fed to the ADC but not to an Op-amp. The details are discussed in the following subsections.

#### 2.2.1. Fixed Resistor Grounding Bleeder Circuit

**Figure 3:**
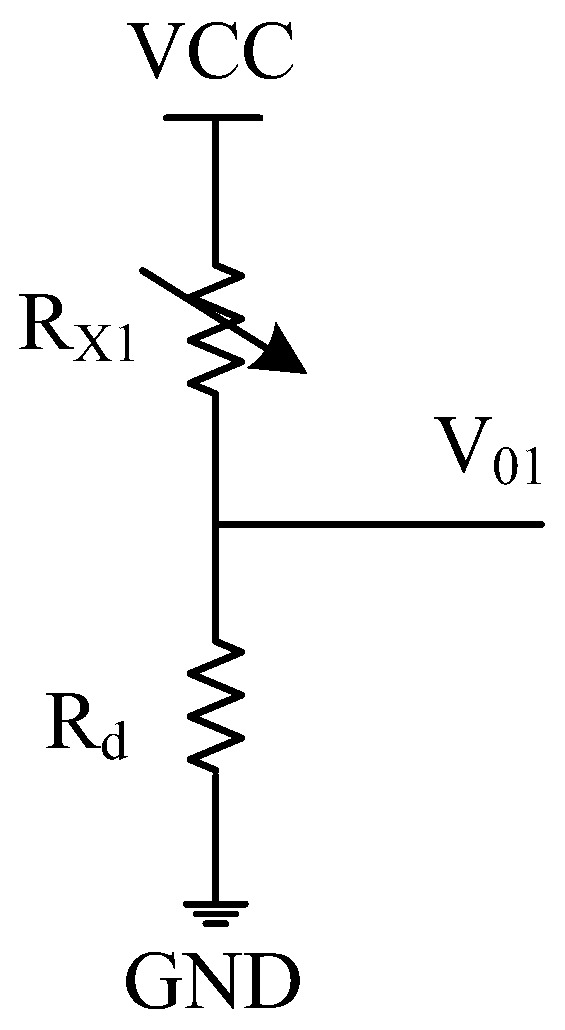
A fixed resistor grounding bleeder circuit.

As illustrated in Figure 3, a fixed resistor grounding bleeder circuit [15] uses a series connection of the fixed resistor *R_d_* and the transducer *R_X_*_1_. *V*_01_ is the output of this resistive voltage divider.

Given $V_{CC}$, the following relationships can be obtained:

(8) $$V_{0}{}_{1}=\frac{R_{d}}{R_{d}+R_{X1}}\times V_{CC}$$

(9) $$\left(\frac{R_{d}}{R_{d}+{R_{1}}^{\prime }}\times V_{CC}-\frac{R_{d}}{R_{d}+{R_{2}}^{\prime }}\times V_{CC}\right)\leq V_{ADC}$$

(10) $$\frac{R_{d}}{R_{d}+{R_{1}}^{\prime }}\times V_{CC}\leq V_{ADC}$$

(11) $$\frac{R_{d}}{R_{d}+{R_{2}}^{\prime }}\times V_{CC}\leq V_{ADC}$$

where *V_ADC_* is the reference voltage of the ADC; $R_{X1}\in [{R_{1}}^{\prime },{R_{2}}^{\prime }]$, ${R_{1}}^{\prime }$, and ${R_{2}}^{\prime }$ are the respective lower and upper limits of the transducer resistance *R_X_*_1_.

For an ADC circuit, the *V*_01_ variation is within the range of the ADC [20,21]. If the ADC has *N* bits and *D_o_* is the ADC output, then:

(12) $$\frac{V_{01}}{V_{ADC}}=\frac{D_{o}}{2^{N}-1}$$

From Equations (8) and (12), the transducer resistance *R_X_*_1_ can be estimated by:

(13) $$R_{X1}=\frac{\left(2^{N}-1\right)\times R_{d}\times V_{CC}}{V_{ADC}}\times \frac{1}{D_{o}}-R_{d}$$

where *N* is the number of bits of the ADC.

**Figure 4:**
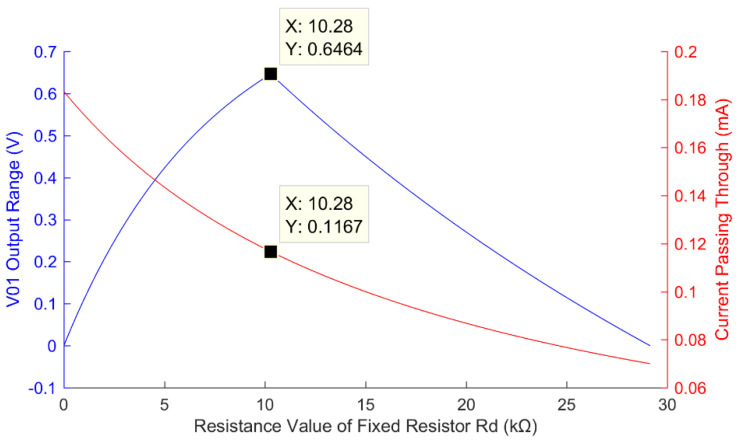
Relationship between the output range (the blue line) and current loss (the red line) of the fixed resistor grounding bleeder circuit and the resistance value of the fixed resistor.

As illustrated in Figure 3, the range of *V*_01_ is between 0 and *V_ADC_*. For our designed smart thermometer, if its temperature measurement range is over 25~50 °C, the corresponding resistance range will be 18~51 kΩ, or ${R_{1}}^{\prime }$ = 18 kΩ and ${R_{2}}^{\prime }$ = 51 kΩ. If the ADC reference voltage *V_ADC_* = 1.2 V, external DC supply voltage $V_{CC}$ = 3.3 V, and *R_X_*_1_ = 18 kΩ, Figure 4 shows the simulation results of the relationship between the output range and current loss of the fixed resistor grounding bleeder from Equations (9)–(11).

From Figure 4, it can be seen that with the increase in the fixed resistor *R_d_*, the output range of *V*_0__1_ increases. *R_d_* = 0 kΩ corresponds to *V*_0__1_ being grounded. When *R_d_* = 10.28 kΩ, *V*_0__1_ reaches its maximum output. When *R_d_*> 10.28 kΩ, the upper limit of *V*_0__1_ will be greater than the reference voltage *V_ADC_* of the ADC. The valid output range of *V*_0__1_ will be the difference between *V_ADC_* and the lower limit of *V*_0__1_. When the lower limit of *V*_0__1_ is equal to *V_ADC_*, the output *V*_0__1_ will go beyond the range of the ADC. In other words, when *R_d_*= 10.28 kΩ, the measuring range of the ADC is the largest and the conversion accuracy is optimal.

Figure 4 also shows the change of the current in mA with respect to the change of *R_d_*. It is seen that the current decreases with the increase of *R_d_*. The measurement accuracy depends on the resolution of the conversion circuit; at the optimum resolution when *R_d_*= 10.28 kΩ, the current is 0.12 mA.

#### 2.2.2. The Proposed Low-Power Circuit

**Figure 5:**
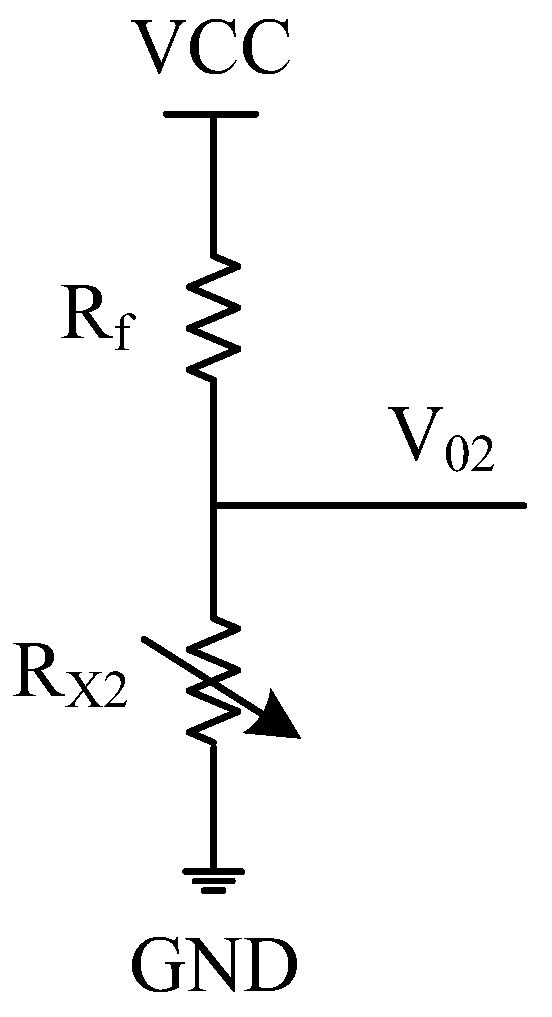
Transducer grounding bleeder circuit.

Figure 5 shows the proposed low-power circuit. It is a transducer grounding bleeder circuit, where *R_f_* is in series connection with the transducer *R*_X__2_.

With respect to $V_{CC}$, the following equations can be obtained:

(14) $$V_{02}=\frac{R_{X2}}{R_{f}+R_{X2}}\times V_{CC}$$

(15) $$\frac{{R_{4}}^{\prime }}{R_{f}+{R_{4}}^{\prime }}\times V_{CC}-\frac{{R_{3}}^{\prime }}{R_{f}+{R_{3}}^{\prime }}\times V_{CC}\leq V_{ADC}$$

(16) $$\frac{{R_{4}}^{\prime }}{R_{f}+{R_{4}}^{\prime }}\times V_{CC}\leq V_{ADC}$$

(17) $$\frac{{R_{3}}^{\prime }}{R_{f}+{R_{3}}^{\prime }}\times V_{CC}\leq V_{ADC}$$

where $R_{X2}\in [{R_{3}}^{\prime },{R_{4}}^{\prime }]$, ${R_{3}}^{\prime }$_,_ and ${R_{4}}^{\prime }$ are the respective lower and upper limits of the transducer resistance *R_X_*_2_.

From Figure 5, the current through this circuit decreases with the increase in *R*_X__2_, or *V*_0__2_ increases with the increase in *R_X_*_2_.

If the ADC has *N* bits and *D_o_* is the ADC output, then:

(18) $$\frac{V_{0}{}_{2}}{V_{ADC}}=\frac{D_{o}}{2^{N}-1}$$

From Equations (14) and (18), *R_X_*_2_ can be obtained as

(19) $$R_{X2}=\frac{R_{f}\times V_{ADC}\times D_{o}}{\left(2^{N}-1\right)\times V_{CC}-V_{ADC}\times D_{o}}$$

**Figure 6:**
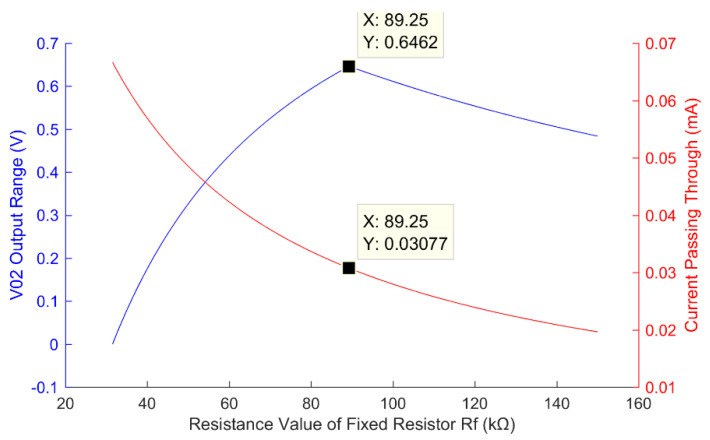
Relationship curves between the output range (blue line) and circuit loss (red line) of the transducer grounding bleeder circuit and the resistance value of the fixed resistor.

In our designed smart thermometer, its resistance range is 18~51 kΩ, or ${R_{3}}^{\prime }$ = 18 kΩ and ${R_{4}}^{\prime }$ = 51 kΩ, and the measurable temperature range is 25~50 °C. Taking *R_X_*_2_ = 18 kΩ as an example, Figure 6 shows the simulation results of the relationship between the output range and the circuit loss of the transducer grounding bleeder circuit using Equations (15)–(17).

It is seen from Figure 6 that with the increase in the resistance of the fixed resistor *R_f_*, the lower limit of *V*_0__2_ is less than *V_ADC_*, and the valid output range of *V*_0__2_ is the difference between *V_ADC_* and the lower limit value of *V*_0__2_. When *R_f_* = 89.25 kΩ, the upper limit of *V*_0__2_ equals *V_ADC_*, and the output of *V*_0__2_ reaches its maximum value. When the lower limit of *V*_0__2_ is equal to *V_ADC_*, the valid output *V*_0__2_ = 0. When *R_f_* > 89.25 kΩ, the output of *V*_0__2_ decreases. In other words, when *R_f_* = 89.25 kΩ, the measuring range of ADC is the largest and the conversion accuracy is the highest. Furthermore, it is seen in Figure 6 that the current decreases with the increase in *R_f_*. At the optimal resolution, the current is 0.03 mA. Therefore, the power consumption of the proposed low-power circuit is lower than the fixed resistor grounding bleeder circuit.

## 3. Development of a Smart Clinical Thermometer

The improved low-power resistive transducer circuit in Section 2.2.2 is implemented for the development of a smart thermometer product for human body temperature monitoring in real-time [22,23,24]. The objective is to develop a new smart clinical thermometer product that is cheaper with lower power consumption. If the temperature exceeds a threshold or the temperature trend is abnormal, the backstage system can provide an early warning of the abnormal situation [25,26].

In this smart thermometer project, because the Bluetooth wireless communication protocol has the advantages of low power consumption, easy use, and a stable link, it is adopted for the data wireless transmission. The Bluetooth chip DA14580 (from Dialog Semiconductor) is selected in this project [27], which has current consumptions of 3.4 mA when sending and 3.7 mA when receiving. It has dedicated hardware for the Link Layer implementation of Bluetooth low-energy and interface controllers to enhance connectivity. The transceiver interfaces with the antenna directly and is fully compliant with the Bluetooth 4.2 standard.

The temperature data is transferred to a computing device (e.g., a cell phone, a computer, a cloud drive) using a general APP with the Bluetooth 4.2 standard. The temperature data can also be transferred to a doctor’s office for health diagnosis.

In accordance with the measurement range of 32~42 °C related to human body temperatures, a 3.7 V lithium-ion battery is used as the power source in this smart thermometer [28]. According to the analysis in Section 2.2.2, the transducer grounding divider conversion circuit is applied in this project, as it not only has a more streamlined circuit design than the bridge conversion circuit (Section 2.1), but also lower power consumption than the fixed resistor grounding divider circuit (Section 2.2.1). An NTC thermistor (model 503ET-87L) is used as the resistive transducer [29], which has a high sensitivity, low cost, and small size, with characteristics of *B*_25/50_ = 3950k ± 1%, zero power resistance *R*_0_ = 29.986~30.016 kΩ, and time response = 3.2 s. Corresponding to the temperature measurement range from 32 to 45 °C, its resistance is between 25.292 and 38.137 kΩ. From Equations (15)–(17) with V_ADC_ = 1.2 V, the optimal resistance of the fixed resistor *R_f_* is determined as *R_f_* = 66.74 kΩ.

**Figure 7:**
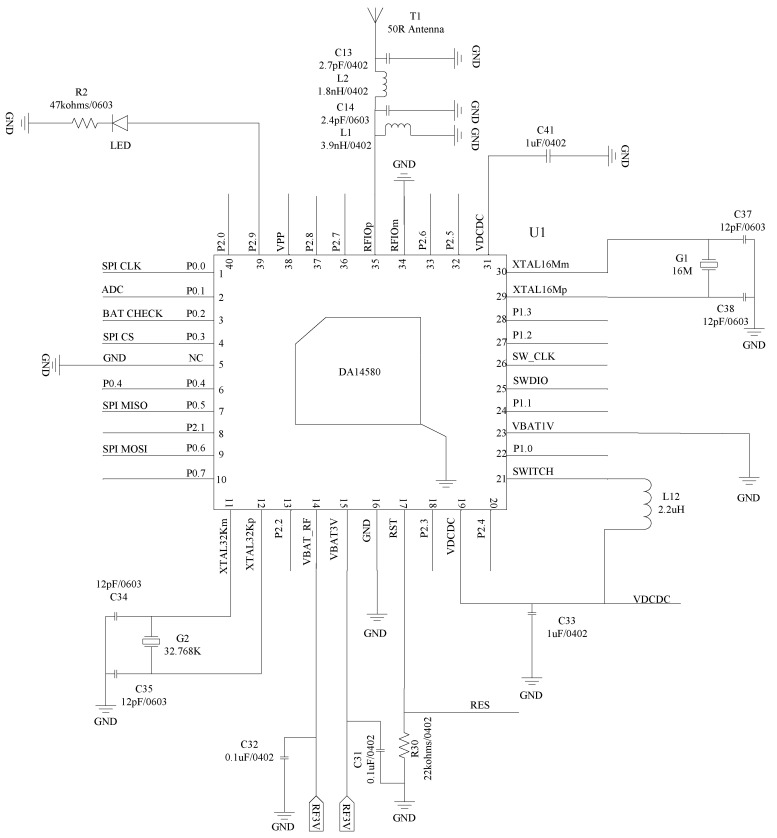
Peripheral circuit of the MCU chip.

The circuit design of the smart thermometer includes the design of the chip peripheral circuit, external flash circuit, temperature conversion circuit, wireless charging circuit, battery power acquisition circuit, and so on. The MCU chip is selected as DA14580 (32-bit ARM Cortex-M0 kernel), with two crystal oscillators at 16MHz (XTAL16M) and 32.768 kHz (XTAL32K), respectively. The 32.768 kHz oscillator is used as the clock of the Extended/Deep Sleep modes. Figure 7 shows the designed peripheral circuit of the MCU.

**Figure 8:**
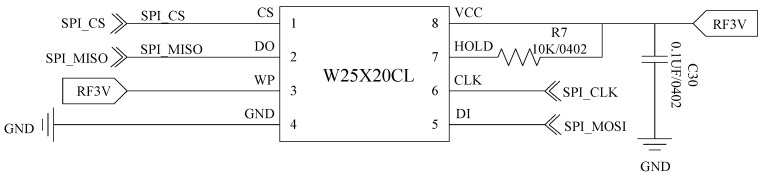
External flash circuit.

The DA14580 chip has a built-in 32 KB One-Time-Programmable (OTP) memory, which can be programmed once only, using an extra flash chip W25X20CL [30]. As shown in Figure 8, the W25X20CL serial flash memory can not only provide an extra storage solution, but also a solution with more flexibility and better performance than using an ordinary serial flash device. An SPI protocol is used for the data communication with the chips.

**Figure 9:**
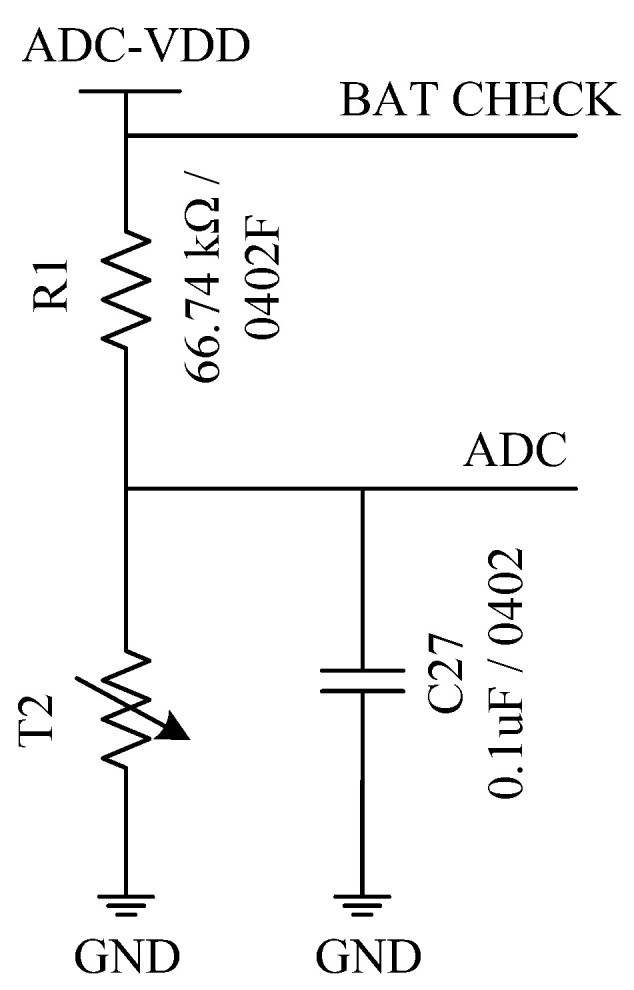
Temperature conversion circuit.

Figure 9 shows the designed transducer grounding divider conversion circuit for temperature conversion. The fixed resistor *R*_1_ is selected as 66.74 kΩ (a nonstandard resistance with a nominal value of 66.5 kΩ in E96 1% precision series). C27 is a 0.1 µF ceramic capacitor that is used to reduce the high-frequency interference caused by the instantaneous change of the load current [31]. The ADC is connected to P0.1 pin of DA14580 in Figure 7 to transmit the voltage of T2 to the ADC. The BAT CHECK is connected to P0.2 pin of DA14580 in Figure 7 to detect the power supply voltage.

**Figure 10:**
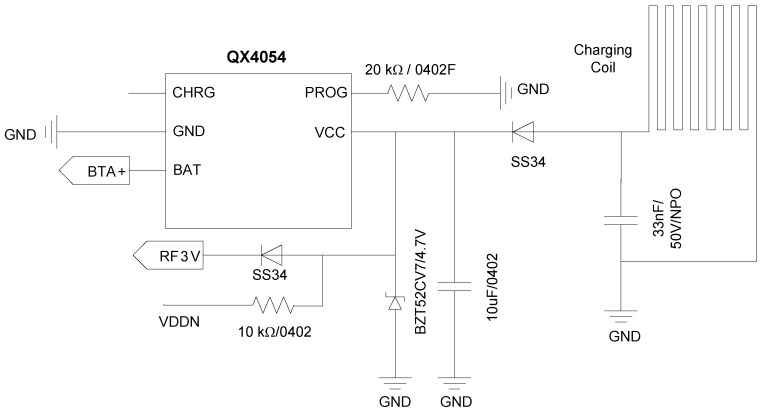
The lithium-ion battery recharging circuit.

To reduce the dimension of the smart thermometer for the convenience of application, a lithium-ion battery (model 401119) is selected as the power source for the circuit, whose battery capacity is 100 mAh. To facilitate application, the battery can be recharged wirelessly. QX4054 (from Qxmd) [32] is selected as the battery recharging chip, which is a constant current and constant voltage linear charging chip. QX4054 has an internal MOSFET structure, which is suitable for portable applications. Figure 10 shows the battery charging circuit. The charging coil and the 33 nF capacitor form an inductance-capacitance (LC) resonant circuit to charge the lithium-ion battery.

**Figure 11:**
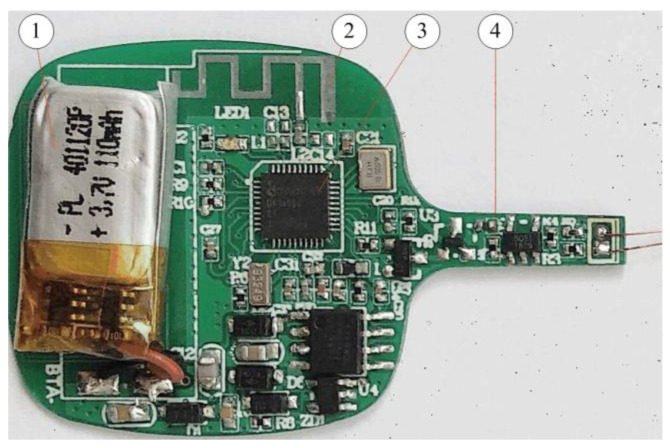
The developed PCB of the smart clinical thermometer: (1) lithium-ion battery, (2) antenna, (3) DA14580 chip, and (4) the fixed resistor *R_f_*.

**Figure 12:**
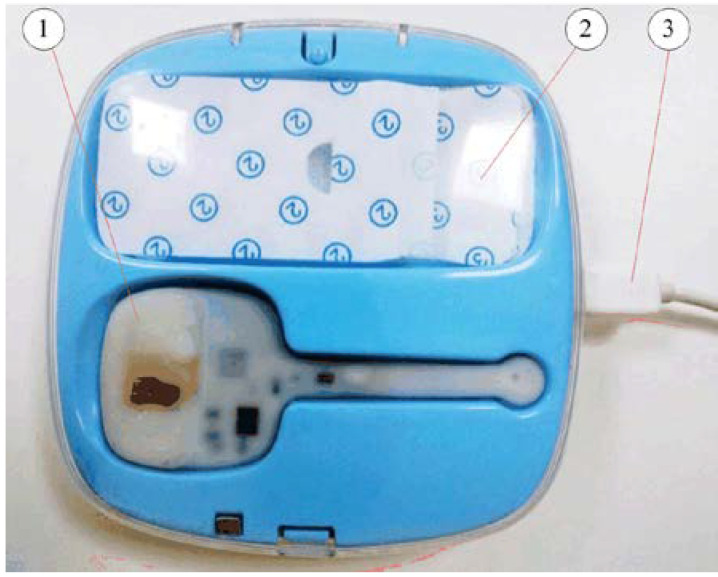
The developed smart thermometer product: (1) the Bluetooth thermometer, (2) the monitor screen, and (3) an extra charging port.

In the PCB design, considering the special design requirements of a small PCB size and high device density, special considerations are undertaken in the PCB layout and wiring design to reduce the influence of distributed inductance, EMC, and crosstalk. The design also involves Bluetooth antennas, charging coils, component layouts, etc. Figure 11 depicts the 2-layer PCB of this developed smart thermometer. Figure 12 shows a prototype of the developed smart clinical thermometer product, where the screen can display the body temperature and other related monitoring information (e.g., system control setup, time period for recording, alarming, Bluetooth transmission set-up).

There are several types of Bluetooth thermometers on the market, such as the Bluetooth thermometer TWJ [33] and Children’s thermometer BLE 4.0 [34]. The main control chip of TWJ is AMICCOM A8105 [35]. Although AMICCOM A8105 does not need an extra flash chip, it is more expensive than DA14580 and W25X20CL as used in this project. In addition, these available thermometers adopt a bridge conversion circuit connected with an Op-amp, which has a higher cost. On the other hand, the main control chip of BLE 4.0, CC2540 [36], requires an extra flash chip and a bridge circuit connected with an Op-amp. Correspondingly, the designed thermometer in this project is cheaper than most of these available thermometers.

## 4. Experimental Tests and Analysis

### 4.1. Testing and Analysis of the Improved Transducer Grounding Bleeder Circuits

**Figure 13:**
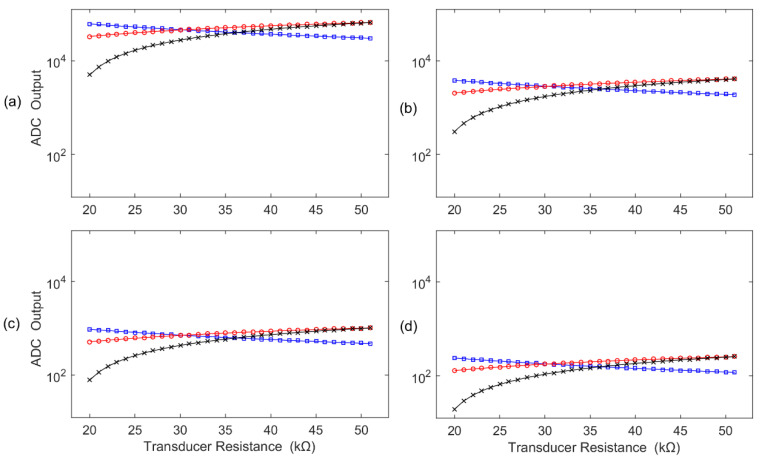
ADC output comparison among three grounding circuits: (1) ×: classical bridge conversion circuit; (2) □: fixed resistor grounding bleeder circuit; (3) ο: transducer grounding bleeder circuit. The results correspond to the (**a**) 16-bit ADC, (**b**) 12-bit ADC, (**c**) 10-bit ADC, and (**d**) 8-bit ADC.

Based on the specific measurement requirements, the optimal resistance value of the fixed resistor can be determined using the method discussed in Section 2.2. Four commonly used ADCs with 8, 10, 12, and 16 bits are considered. Figure 13 shows the relationship between the ADC outputs and the resistance of the resistive transducer *R_X_*. It also demonstrates the comparison results among the three grounding circuits: (1) the classical bridge conversion circuit, (2) the fixed resistor grounding bleeder circuit, and (3) the transducer grounding bleeder circuit. Table 1 summarizes the comparison results of these three circuits.

Based on the above demonstrated results, the following conclusions can be drawn:

(1) The ADC outputs differ with the ADC bits (*N* = 8, 10, 12, and 16 bits).

(2) By using the improved conversion circuit, the output of the transducer grounding divider conversion circuit has a positive correlation with the increase in the transducer resistance, which is better than the fixed resistor grounding divider conversion circuit, which has a negative correlation with the increase in the transducer resistance.

(3) The ADC output of the proposed transducer grounding divider conversion circuit (red line) is also higher than that of the classical bridge conversion circuit (black line).

(4) The resolution of the transducer grounding bleeder circuit is approximately the same as the fixed resistor grounding bleeder circuit, but its power consumption is lower than that of the fixed resistor grounding bleeder circuit.

(5) Given an ADC with specific bits, although the measurement resolution of the thermometer based on the resistive divider conversion circuit could be lower than that based on the classical bridge conversion circuit, its resolution can be improved by using a higher-bit ADC. For example, the measurement resolution of a 12-bit ADC for the resistive divider conversion circuit (i.e., 1.18 × 10^−2^ °C) is about 2 times higher than that of the traditional bridge conversion circuit using a 10-bit ADC (i.e., 2.55 × 10^−2^ °C). Meanwhile, when the ADC has 12 bits or higher, its measurement resolution can meet the requirements of this clinical thermometer while its circuit is more streamlined and is cheaper in cost.

### 4.2. Test and Analysis of the Smart Thermometer System

**Figure 14:**
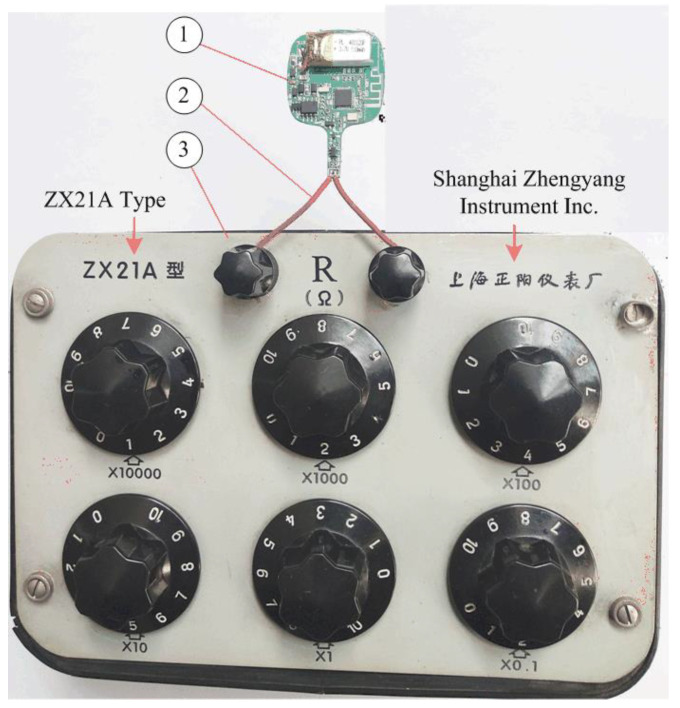
Experimental set-up for smart thermometer testing: (1) the developed smart thermometer PCB, (2) leading wires, and (3) a DC resistor device.

Figure 14 shows the experimental set-up used to examine the accuracy of the developed smart thermometer product. The NTC thermistor is replaced by a DC resistor device. In testing, the leading wires should be as short as possible to reduce the influence of the wire resistance on the experiment results.

**Figure 15:**
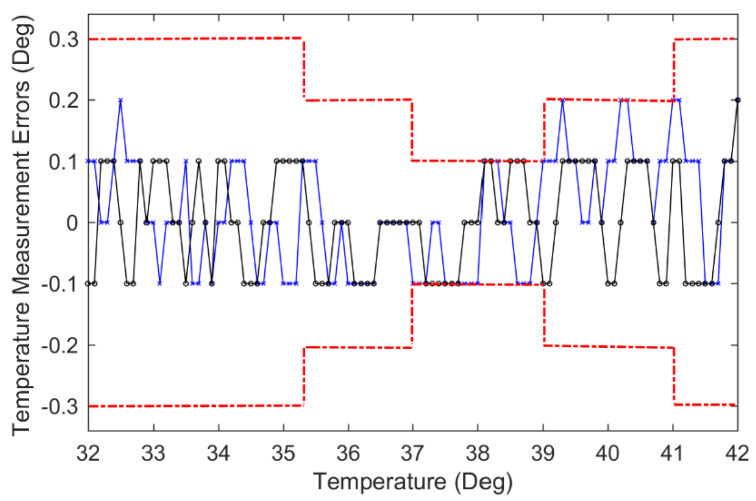
Test result comparison: ×—blue line: upstroke errors; ο—black line: downstroke errors; red dotted line: allowable error thresholds by the related regulations in China.

The experimental tests cover the general temperature range of 32~42 °C. The one-to-one correspondence between the body temperature value and the theoretical resistance value can be established by calibration. Figure 15 demonstrates the experimental results. The upstroke temperature and the downstroke temperature are the mean values over 10 simulation tests, where upstroke refers to the change in the output as the input increases and the downstroke denotes the change in the output as the input decreases. All of the tests and measurements have followed the regulations in accordance with the National Standards of Clinical Electronic Thermometer GB/T 21416 in China. The temperature measurement range and the maximum permissible errors of the thermometers are listed in Table 2. It can be seen that the tested temperature errors in Figure 15 are less than those regulated by GB/T 21416 in China, thus the developed smart clinical thermometers can meet the requirements (they have become commercial products in China).

## 5. Conclusions

A new type of clinical Bluetooth thermometer product was developed in this work for real-time clinical body temperature measurement and monitoring. An improved low-power resistive transducer circuit was proposed to simplify the circuit design and to reduce the product cost and power consumption. A quantitative analysis method was proposed to optimize the circuit parameters to improve system performance. The effectiveness of the improved conversion circuit technology was verified by experimental tests. The test results show that the transducer grounding divider conversion circuit outperforms the fixed resistor grounding divider conversion circuit and the classic bridge circuit. In addition, the developed smart thermometer can meet the requirements of clinical measurement applications, with a more streamlined circuit design, lower cost, and lower power consumption.

## Figures and Tables

**Table 1:** Comparison of the three circuits.

ADC Bits	Classical Bridge Conversion Circuit		Fixed Resistor Grounding Bleeder Circuit		Resistive Transducer Grounding Bleeder Circuit	
	Conversion Range	Measurement Resolution	Conversion Range	Measurement Resolution	Conversion Range	Measurement Resolution
8	255	±1.02 × 10^−1^ °C	138	±1.90 × 10^−1^ °C	138	±1.89 × 10^−1^ °C
10	1021	±2.55 × 10^−2^ °C	551	±4.73 × 10^−2^ °C	551	±4.72 × 10^−2^ °C
12	4088	±6.11 × 10^−3^ °C	2205	±1.18 × 10^−2^ °C	2205	±1.17 × 10^−2^ °C
16	65,420	±4.22 × 10^−4^ °C	35,280	±7.38 × 10^−4^ °C	35,288	±7.36 × 10^−4^ °C

**Table 2:** Thermometers’ maximum permissible errors by GB/T 21416 in China.

Temperature Display Range (°C)	Maximum Allowable Errors (°C)
Lower than 35.3	±0.3
35.3~36.9	±0.2
37.0~39.0	±0.1
39.1~41.0	±0.2
Higher than 41.0	±0.3

## Data Availability

The study did not report any data.
